# The climate crisis is here: a primer and call to action for public health nutrition researchers and practitioners in high-income countries

**DOI:** 10.1017/S1368980022002427

**Published:** 2022-11-04

**Authors:** Brooke M Bell

**Affiliations:** 1 Department of Chronic Disease Epidemiology, Yale School of Public Health, Yale University, New Haven, CT, USA; 2 Division of Agriculture, Food, and Environment, Friedman School of Nutrition Science and Policy, Tufts University, 150 Harrison Avenue, Boston, MA 02111, USA

**Keywords:** Sustainable diets, Plant-based diets, Food systems, Climate change, High-income countries

## Abstract

Dietary behaviours and the food systems in which they occur have a significant impact on climate change. The 2022 Intergovernmental Panel on Climate Change (IPCC) reports and other major climate reports have identified population-level dietary shifts towards balanced, sustainable healthy diets as an important mitigation (i.e. prevention) solution for climate change. Thus, public health nutrition researchers and practitioners have a crucial role to play in combatting the climate crisis. They have the content expertise, interdisciplinary training and technical skills needed to facilitate wide-scale dietary behaviour changes at multiple levels of influence and ultimately improve both human and planetary health. This commentary article: (i) summarises how dietary behaviours and food systems contribute to climate change, with a particular focus on high-income countries; (ii) reviews food-system-related climate change mitigation solutions most relevant to public health nutrition researchers and practitioners; and (iii) identifies key gaps in the literature and future research directions for the field.

The climate crisis is among the greatest public health threats in history, as an increasingly warming planet creates dire health consequences across the globe^(1,2)^. The latest Intergovernmental Panel on Climate Change (IPCC) reports^(3,4)^ released in early 2022 make it clear that public health nutrition researchers and practitioners play a vital role in preventing climate catastrophe.

Dietary behaviours and the food systems in which they occur have a significant impact on climate change^(4)^, with the largest food-related greenhouse gas (GHG) emissions per capita coming from high-income countries such as the USA, Canada and Australia^(5)^. Climate research provides us with impactful solutions spanning the food system, but political, economic, social, behavioural, and cultural obstacles and concerted obstruction from the for-profit agriculture and food industry stymy the solutions’ implementations^(6)^. Public health nutrition researchers and practitioners have the content expertise, technical skills and interdisciplinary training needed to collaborate across disciplines, overcome these structural obstacles and successfully facilitate wide-scale dietary behaviour changes that are beneficial for people and our planet.

This commentary article: (i) summarises how dietary behaviours and food systems contribute to climate change, with a particular focus on high-income countries; (ii) reviews food-system-related climate change mitigation solutions that are most relevant to public health nutrition researchers and practitioners; and (iii) identifies key gaps in the literature and future research directions for the field.

## Role of diet and food systems

Our dietary habits and the food system are major contributors to climate change and pollution. One IPCC report estimates that the global food system (including agriculture and related land use) accounts for 23–42 % of global GHG emissions^(4)^. Limiting the global temperature increase to 1·5 degrees Celsius (equivalent to 2·7 degrees Fahrenheit) set by the Paris Climate Agreement requires deep reductions in food-related emissions^(7)^. Large-scale transformations throughout the food supply chain, including demand-side mitigation and behavioural changes, must occur for us to avoid catastrophic climate change.

### Food production

The largest proportion of food-related GHG emissions (about 39 %) occurs on farms during the food production stage^(5)^. The production of animal products – beef and veal in particular^(8)^ – contributes the most farm-stage emissions through the ruminant process of *enteric fermentation* (i.e. animal burps that expel methane) and animal manure that emits methane and nitrous oxide^(9)^.

The problem’s scope is immense, as the global cattle population reached approximately 1·1 billion in 2022 (primarily produced in the USA)^(10)^. However, meat and dairy products only provide approximately one-third of the world’s protein supply compared with plant-based proteins^(11)^, which also take up substantially less land^(12)^. This disproportionate agricultural land use for animal-based proteins will be unsustainable in the coming decades as the global population rises past 9 billion by 2050^(13)^.

The second highest farm-stage emissions come from the over-application of synthetic nitrogen-based fertilisers^(9)^, which can lead to excess nitrogen that is emitted into the atmosphere as nitrous oxide or washed out of the soil into waterways^(14)^.

The third highest farm-stage emissions come from rice cultivation^(9)^. Rice grows in water-flooded rice paddies, which cultivate bacteria that emit large quantities of methane^(15)^. However, when compared with animal-based foods, rice production has overall less environmental impact per ton of protein consumed globally^(16)^.

### Land use and land use change activities

The second largest proportion of food-related GHG emissions (about 32 %) comes from land use and land change activities^(5)^, such as deforestation to convert forest into pastureland to raise beef and other livestock or into cropland^(17)^. The FAO estimates agricultural expansion drives almost 90 % of global deforestation^(18)^.

This is critical because forests are a major *carbon sink*: trees absorb carbon dioxide from the atmosphere and store carbon in their trunks, branches and roots. Deforestation releases the stored carbon into the atmosphere, thus contributing to GHG emissions and biodiversity loss^(19)^, which threatens to displace thousands of species from their habitats^(20)^.

### Other supply chain activities

Other food system stages (food processing, food distribution, food consumption and end-of-life food disposal) account for the remaining proportion of food-related GHG emissions (about 29 %)^(5)^. GHG emissions from food transportation accounts for less than 10 % of emissions for most food products^(21)^, suggesting that *what* you eat is substantially more important for climate change mitigation than *where* your food comes from.

## Mitigation solutions

Given the substantial impact of the *types of foods* we consume on GHG emissions, the recent IPCC reports and other major climate reports have identified population-level dietary shifts towards balanced, sustainable healthy diets as an important mitigation (i.e. prevention) solution for climate change^(4,16,22,23)^.

There is no single definition or description of *sustainable diets*; however, the FAO suggests that sustainable diets should serve multiple functions: they should promote health and wellbeing; have low environmental impact; be accessible, affordable, safe, and equitable; and be culturally acceptable^(22)^. This looks different in different populations, contexts and cultures, but broadly, sustainable diets can be characterised as diets rich in plant-based foods, such as fruits, vegetables, whole grains, legumes, nuts, and unsaturated oils, and contain low amounts of animal-based foods, refined grains, added sugars, and unhealthy fats^(24)^.

High-income countries consume a disproportionate amount of meat and dairy products compared with the rest of the world^(25)^, so transitioning towards largely plant-based, environmentally sustainable diets (e.g. flexitarian, vegetarian and vegan), including deep reductions in or elimination of beef intake, in high-income countries^(26)^ can have a major impact on mitigating climate change^(24,27)^. Importantly, these dietary patterns have major benefits for human health^(28,29)^, can be nutritionally adequate^(30)^, and can readily meet or exceed recommended protein intake^(30)^. Even if climate change were not an issue, transitioning towards plant-based diets in high-income countries would still be a major public health imperative due to these immediate health benefits.

## A call to action

To achieve the Paris Climate Agreement goals, the world needs to halve GHG emissions by 2030^(31)^. One IPCC report estimated that nearly half of food-related GHG emissions by 2050 could be mitigated through demand-side changes^(4)^. Bold and swift action is needed to transform society such that healthy and sustainable food choices are convenient, affordable, and, ultimately, the default choice for consumers. Incremental progress will fail – we need large-scale solutions implemented at every level of influence to accomplish this transformation. Moreover, these proposed dietary shifts need to happen alongside other changes to the food and agricultural system, such as the proposed and in-progress improvements in agricultural production practices and technologies^(32)^, diversifying the protein sources for human consumption and animal feed (e.g. tofu, cultured meats and plant-based milk)^(33)^, and reductions in food waste^(4)^. Furthermore, collaboration across disciplines and stakeholders are needed to successfully translate research into policy.

While the *type* of dietary changes (e.g. increased intake of plant-based foods) needed to mitigate climate change have been proposed, and in some cases, implemented on a small scale^(34,35)^, insufficient progress has been made on actually changing population-level dietary habits given the urgency of the climate crisis. Public health nutrition A call to action for public health nutrition researchers and practitioners have studied and successfully intervened on dietary habits for decades and thus can provide valuable expertise on this topic.

## Past and current advances

An abundance of literature has proposed and investigated food-related climate change mitigation solutions at multiple levels of influence (e.g. individual, environment, policy, etc.)^(35–37)^. At the individual level, providing information about and access to sustainable healthy foods can influence food choice and demand^(38)^. Additionally, framing climate change as a public health issue and highlighting the health ‘co-benefits’ of climate action may be an effective strategy to enhance public engagement^(39)^.

Physical environment-level interventions that ‘nudge’ individuals towards climate-friendly food products can include changes to food product positioning, prominence, visibility, availability, portion and/or package size^(40)^. Sustainability-related food labels in food markets can potentially encourage sustainable food choices and ultimately influence market forces^(41)^.

However, changes to individual-level eating behaviour or the physical food environment alone are not sufficient to produce the needed reductions in food system GHG emissions. We must prioritise policy actions, which arguably can produce the largest and widest impact. For instance, public procurement standards, which guide the purchase of food products for government- and state-owned enterprises, such as public schools, hospitals and prisons, can be amended such that all procured food products are required to be demonstrably environmentally sustainable^(42)^. Fiscal measures, such as taxes on animal-based foods, subsidies for plant-based foods and foreign trade policies (e.g. tariffs and duties), can be introduced to: incentivise the production, sales and consumption of climate-friendly food products; disincentivise emissions-intensive food products^(35,43)^; and potentially address well-deserved concerns about the current affordability of plant-based diets^(44)^. National dietary guidelines and associated programmes (e.g. government food assistance programmes) can be modified such that they incorporate values of environmental sustainability^(45)^, as some countries including Canada, Switzerland and Sweden have already done.

Regulations and polices targeted at the agricultural and food industry are also necessary to implement these mitigation solutions. Agribusiness (also referred to as ‘Big Ag’) spends millions of dollars lobbying against climate policies and uses its influence over governments to sabotage progress on climate change in order to protect its profits^(6)^. National governments can implement various policies to weaken this influence, including breaking up the agri-business monopolies (e.g. Bayer-Monsanto and Tyson Foods), placing a moratorium on future mergers^(46)^, ending subsidies for factory farms, shifting farm subsidies towards small family farmers and strengthening regulations to prevent deforestation in critical areas like the Brazilian Amazon^(47)^.

Table 1 contains examples of food-related mitigation solutions targeted at different levels of influence that have been proposed or implemented in the real world.

## Future directions

While accumulating evidence makes it clear that changes to dietary behaviours and the food production system in high-income countries can have substantial impacts on reducing global GHG emissions, what is less clear is how to encourage and enact these changes both at the individual and structural levels.

Tackling the proposed dietary shifts will require public health nutrition researchers and practitioners to navigate the complex and interconnected social, cultural, economic, and political systems that eating behaviours take place in. Interdisciplinary research will be necessary to accomplish our goals – we must collaborate with policy-makers, economists, sociologists, behavioural scientists, healthcare professionals, food system actors (e.g. food producers, food retailers, etc.), advocacy groups, community leaders and many others, to successfully facilitate population-level dietary shifts.

## Key gaps in the literature

Several areas of interdisciplinary research are urgently needed to address key gaps in the literature:

1. Determining the most effective solutions that encourage the intake of plant-based and low-emissions foods beyond fruits and vegetables (which have received the majority of attention thus far), such as whole grains, legumes^(48)^ and alternative proteins.
2. Testing various structural-level solutions (i.e. changes to physical environment and policies) that promote the consumption of plant-based foods among the general population rather than easier-to-reach populations (e.g. university students)^(36)^.
3. Investigating how social relationships, sociocultural norms and social movements can be leveraged to promote the consumption of plant-based foods.
4. Identifying strategies to quickly and efficiently scale up the implementation of interventions in the real-world that have been successful in small, controlled studies.
5. Determining the best set or combination of strategies and policies (i.e. ‘policy packages’) that can most effectively facilitate dietary behaviour changes and reduce food-related GHG emissions in both (i) the short-term and (ii) the long-term.
6. Investigating the role of the agriculture and food industry in obstructing climate progress and identifying effective solutions that overcome this obstruction.
7. Advancing, consolidating and validating the methodologies used to assess and model environmentally sustainable dietary behaviours.
8. Implementing community-based research approaches (i.e. actively engaging and collaborating with community members and leaders), which may produce interventions and other strategies that are more relevant and culturally acceptable to the community – and thus may be more effective.

### Structural solutions

Finally, powerful and intersecting structural factors are key drivers of food, health and climate inequities^(49)^. Therefore, overarching solutions that address these structural factors are also needed to improve diets and reduce these inequities. We must investigate solutions that address structural inequities (e.g. income inequality, structural racism, housing and education opportunities)^(50)^, which may additionally improve the affordability and accessibility of sustainable diets and contribute to food, health, and climate equity.

## Conclusion

Climate change and its current and future effects on population and planetary health is an urgent, complex issue that requires massive societal and behavioural shifts, including in food systems and dietary behaviours. Implementing solutions will require public health nutrition researchers and practitioners to expand the types of data, methods, theories, interventions and scholarly collaborations with which the field is most familiar.

These are incredibly substantial tasks, but we can begin to accomplish these tasks by: critically thinking about how to incorporate a ‘climate lens’ into our work; learning about the climate change mitigation efforts already underway at our organisations (or starting these efforts if not already being done); reaching out to community partners and stakeholders that are engaged in climate change mitigation and climate justice efforts; and brainstorming with current and new collaborators on work that incorporates this climate lens.

We must rise to the occasion and take on these unprecedented and challenging tasks. Dietary shifts are insufficient by themselves to solve the entire climate crisis; however, they are needed to reach our climate targets and thus can be an important contribution by public health nutrition researchers and practitioners that ultimately improves both human and planetary health.

## Figures and Tables

**Table 1:** Examples of food-related solutions (i.e. ‘interventions’) that have been proposed or implemented in the real world

Level of influence	Strategies used	Location of intervention	Description of intervention	Links
Individual level	Information provision; change social norms	Social media (online)	In 2022, Grammy Award-winning Kelly Rowland used her social media platform (TikTok) to encourage viewers to switch from animal-based to plant-based milk products.	https://www.tiktok.com/@kellyrowland/video/7083136908120919342
	Change social norms; discourage	Outdoors	In 2020, Oscar-winner Joaquin Phoenix was featured in an ‘anti-meat’ billboard in Los Angeles sponsored by non-profit Los Angeles Animal Save.	
Environment level	Information provision; change to the food environment (food labels); behavioural nudge	Food market	British multinational consumer goods company Unilever announced in 2021 that it was working on introducing carbon labelling for all of its food products.	https://www.unilever.com/news/news-search/2021/carbon-footprints-now-its-personal/
	Change to the food environment; increase choices	Food market	In 2018, the first all-vegan food market called ‘VeganSpace’ opened in Seoul, South Korea.	https://www.instagram.com/p/BkzKoE1nE26/
	Change to the food environment; change to the default; behavioural nudge	School	Food supplier Sodexo, which supplies more than 1000 US universities and colleges, aims to transition 33% of its campus menu offerings to plant-based by 2025.	
	Change to the food environment; restrict choices	Concert (event)	In California, the 2022 Cruel World Music Festival’s food lineup was entirely vegetarian and vegan.	
	Change to the food environment; restrict choices	Meeting (event)	In March 2022, the UK’s Oxfordshire County Council announced that it would only serve plant-based food at council catered events moving forward.	https://news.oxfordshire.gov.uk/plant-basedfood/
	Change to the food environment; restrict choices	Restaurant	Burger King’s Leicester Square (London) flagship restaurant went 100% meat-free from March to April 2022.	https://www.burgerking.co.uk/meat-freerestaurant
	Change to the food environment restrict choices	Restaurant	Eleven Madison Park, a fine dining restaurant in Manhattan, shifted to a completely plant-based menu when it reopened in 2021 after the Covid-19 pandemic restrictions were lifted.	https://www.elevenmadisonpark.com/ourrestaurant/
	Change to the food environment; change to the default; subsidise/incentivise	Restaurant	Starbucks coffeehouse chain has a surcharge for non-dairy milk (e.g. soya, almond, oat, etc.). Alternatively, other popular coffeehouse chains like Panera Bread, Philz Coffee, Blue Bottle Coffee and Pret a Manger provide dairy-free milk for no extra charge. As of 2022, Blue Bottle Coffee uses oat milk, instead of cow’s milk, as the default option at its US café locations.	https://blog.bluebottlecoffee.com/posts/oatmilk-by-default
	Change to the food environment; increase choices	Airplane	As of March 2022, Delta Airlines now offers plant-based burgers and lamb meatballs for Delta One and First Class customers on select flight menus.	https://news.delta.com/impossible-foodsfeatured-new-plant-based-menu-optionsdebut-onboard-delta
	Change to the food environment; change to the default	Workplace	World Resources Institute’s Cool Food programme helps businesses and organisations cut the climate impact of the food they serve. Organisations can take the Cool Food Pledge, which helps them commit to and achieve a target of reducing the GHG emissions associated with the food they serve by 25 %.	https://www.wri.org/initiatives/cool-food-pledge
	Change to the food environment; increase choices	Food market	In 2020, Tesco, the UK’s largest supermarket, set a 5-year goal to increase sales of plant-based proteins by 300 %.	https://www.tescoplc.com/news/2020/tescocommits-to-300-sales-increase-in-meatalternatives/
Policy level	Subsidise/incentivise	Food market	The Double Up Food Bucks programme gives extra benefits to purchase fruits and vegetables to recipients of US SNAP benefits in dozens of US states.	https://doubleupamerica.org/
	Discourage	Food market	In March 2022, a ‘meat tax’ in the Netherlands was proposed by the Dutch Minister of Agriculture; however, it has not been implemented at this time.	https://www.fas.usda.gov/data/netherlandsconcept-meat-tax-under-discussionnetherlands
	Change to the food environment; increase choices	Food market/restaurant	The Singapore Food Agency (SFA) was the first in the world to issue approval for cultured meats. In 2020, the SFA approved the commercial sale of cell-cultured (also referred to as lab-grown) chicken in the form of chicken bites, produced by the company Eat Just, Inc.	https://www.sfa.gov.sg/docs/default-source/publication/annual-report/sfa-ar-2020-20212c7b8b52e3e84fd193c56d53f42fe607.pdf
	Subsidise/incentivise	Healthcare facility; food market	In June 2022, the US Department of Agriculture (USDA) allocated nearly $40 million to the Gus Schumacher Nutrition Incentive Program (GusNIP) Produce Prescription Program. Produce Prescription (or ‘Produce Rx’) projects funded by this programme provide financial and non-financial incentives (often in the form of a fruit and vegetable ‘prescription’) to income-eligible individuals that allow them to increase the procurement and consumption of fruits and vegetables.	https://www.nifa.usda.gov/grants/fundingopportunities/gus-schumacher-nutritionincentive-program-produce-prescription
	Change to the food environment; restrict choices	School	France’s Climate and Resilience Law, adopted in 2021, requires that school cafeterias offer vegetarian menus at least once a week.	https://www.legifrance.gouv.fr/jorf/id/JORFTEXT000043956924
Government level	Information provision	Government websites (or print media)	Countries including Canada, Switzerland, Sweden, Norway and Brazil have all recently incorporated environmental sustainability within their national dietary guidelines	
